# Supplementary material for: Comparison of the Diagnostic Performance of Deep Learning Algorithms for Reducing the Time Required for COVID-19 RT–PCR Testing
Source: Viruses. 2023 Jan 22;15(2):304. doi: 10.3390/v15020304 (PMC9966023; doi:10.3390/v15020304)
Supplement: Supplementary file 1 [file viruses-15-00304-s001.zip › viruses-2014115-supplementary.pdf]

Supplementary table S1. Diagnostic performance of DL models in five different algorithms using the test dataset

	Model 10			Model 20		
	Sensitivity, %	Specificity, %	AUROC, %	Sensitivity, %	Specificity, %	AUROC, %
RNN	90.37	68.52	79.44	97.04	86.67	91.85
LSTM	96.67	69.63	83.15	97.78	86.30	92.04
Bi-LSTM	96.67	73.70	85.19	97.04	89.26	93.15
GRU	95.56	72.96	84.26	97.41	87.41	92.41
Transformer	88.52	67.78	78.15	94.44	89.26	91.85

RNN, recurrent neural network; LSTM, long short-term memory; GRU, gated recurrent unit; Bi-LSTM, bidirectional long short-term memory; AUROC, area under the receiver operating characteristic; DL, deep learning

Supplementary table S2. The effect of the prevalence on the diagnostic performance of each DL algorithm

	Model 10			Model 20		
	PPV, %	NPV, %	Accuracy, %	PPV, %	NPV, %	Accuracy, %
RNN	13.13	99.27	69.61	27.70	99.82	87.19
LSTM	14.35	99.75	70.98	27.30	99.86	86.87
Bi-LSTM	16.21	99.76	74.85	32.23	99.83	89.65
GRU	15.68	99.68	74.09	28.93	99.84	87.91
Transformer	12.63	99.12	68.81	31.64	99.67	89.52

PPV, positive predictive value; NPV, negative predictive value; RNN, recurrent neural network; LSTM, long short-term memory; GRU, gated recurrent unit; Bi-LSTM, bidirectional long short-term memory; AUROC, area under the receiver operating characteristic; DL, deep learning
